# Rural Zambian Oral Health Transition: A Long-Term Retrospective Examination of an Outreach Program’s Progress and Impact

**DOI:** 10.5334/aogh.4179

**Published:** 2023-10-09

**Authors:** John P. Morgan, Olivia N. Marino, Matthew Finkelman, Carlos Fernando Mourão, Felicitas S. Flubinda

**Affiliations:** 1 Tufts University School of Dental Medicine, US; 2 Dental Training School, Lusaka, Zambia

**Keywords:** Oral Health, Community-Based Participatory, Research Health Promotion, Rural Health Services, Dental Caries

## Abstract

**Objective:**

This study aimed to (a) describe an annual prevention-focused, community-based oral health outreach program in rural Zambia, (b) assess its oral health outcomes using demographic and oral health variables, and c) identify milestones resulting from program activities.

**Methods:**

A retrospective analysis of demographic and oral health data from a single site between 2007–2014 and 2018–2019 was conducted. Demographic variables included sex and age, while clinical outcomes encompassed pain, untreated caries, and treatment urgency. Bivariate and multivariable analyses were performed, adjusting for sex and age categories. Information on community development was obtained from the Ministry of Health and local community representatives.

**Results:**

Data from 5,791 subjects were analyzed. The prevalence of pain, untreated caries, and highest treatment urgency category decreased consistently across year categories. Both bivariate and multivariable analyses showed statistically significant differences in clinical outcomes between year categories (*p* < 0.001). In addition, the percentage of male participants and younger age categories increased during the study period. Key program milestones included the installation of two boreholes for clean water, the development of a local community oral health volunteer program, the establishment of an educational pipeline by the Dental Training School for residents, and the construction of a maternal/oral health center with district and ministry oversight.

**Conclusion:**

The observed decrease in treatment urgency scores, presence of pain, and untreated caries are consistent with the prevention-seeking behavior of program participants. The increasing participation and changing demographic patterns over time suggest a growing demand for oral health services among males and younger individuals. The positive oral health outcomes and development of a maternal child/oral health facility exemplify a program design aligned with community needs and appropriate care delivery.

## Introduction

Oral health problems continue to persist, with oral disease disparities among the most disadvantaged [1,2,3]. Affecting an estimated 3.5 billion people, oral diseases are among the most common noncommunicable diseases globally [4,5]. Although largely preventable, the oral disease burden is rising, particularly in low- and middle-income countries. The unequal distribution of oral health personnel and the lack of appropriate oral health infrastructure limit access to primary oral health services. The high prevalence of untreated oral disease and lack of oral health services result in high demand for essential oral health services globally. Recommendations to reduce the burden of oral disease emphasize comprehensive system changes to shift from traditional curative approaches toward preventive approaches [6].

The term “dental aid organization” (DAO) encompasses different associations of various institutions that share the common aim of providing oral health care aid in underserved communities [5,7,8]. Typically, non-governmental organizations such as DAOs facilitate volunteer dental outreach clinical services to meet dental needs, often in low- and middle-income countries [9,10]. DAO efforts are recognized for the time and resources provided for dental services and the potential to support oral health care [11]. Limited information is available regarding program design and the impact of DAO activity on community health and oral disease burden [11]. Recommended elements of effective oral health aid models include (a) capacity building of the local workforce, (b) improved understanding of local contexts, and (c) alignment and integration of oral health care with local health systems. Also recommended to guide program development are program outcome measures such as health awareness, health-seeking behavior, and patient volumes. Program descriptions incorporating these recommendations into practice are not well documented [7].

In 2006, a rural community in the Southern Province of Zambia invited a team of US-based dental and medical volunteers to assist with the development of oral health services. The remote community had a lack of a road network system, limited transportation options, limited access to clean water, no electricity, no access to developed sanitation, limited health services, and no oral health services. It was also isolated during a four-month rainy season. The health personnel at the Rural Health Post, which serves a community with an estimated catchment area of up to 45,000 people, consisted of one nurse and one clinical officer. The medical and dental volunteers engaged community leaders (chief, headmen, and local government representatives), the Community Health Committee, the District Health Director, and the Dental Training School in conjunction with the Ministry of Health of Zambia to form an oral health collaborative. The resultant oral health outreach program, based on the Basic Package of Oral Care [12], consisted of oral health promotion and education, oral health prevention, including the provision of fluoridated toothpaste and toothbrushes, the provision of atraumatic restorative treatment and oral urgent care. In addition to oral health services at annual outreach programs, the multidisciplinary collaborative provided advocacy and support for the development of a community oral health volunteer program, support for clean water resources, the creation of maternal and child health clinical resources, and the integration of oral health into primary health care services in the community. The current study’s aim was to (a) provide a description of the annual prevention-focused community-based oral health outreach program, (b) explore demographic and oral health variables to assess the program’s oral health outcomes, and (c) identify milestones resulting from program activity and advocacy.

## Materials and Methods

### Study design

This study used clinical assessment data recorded from dental records documenting dental treatment provided at annual oral health outreach programs conducted in rural Zambia. No comparative data for oral health services were available for this community. The authors used a retrospective analysis of repeated cross-sectional data completed for each year of activity from 2007 to 2019. To ensure the data’s quality, the authors followed the Strengthening the Reporting of Observational Studies in Epidemiology (STROBE) checklist [13]. This checklist is a set of guidelines developed to enhance the quality and transparency of reporting observational studies in epidemiology. The outreach project was conducted under the guidance of the Namwala Health District of Zambia with support from the Dental Training School of the Ministry of Health of Zambia. The study was granted exempt status by the Tufts Health Sciences Institutional Review Board under the number (13439).

### Setting

Oral health outreach programs of up to four days’ duration took place at the Rural Health Post centrally located in the subsistence farming community. Tents and mobile dental equipment were used to create dental treatment facilities at the Rural Health Post. Oral screening examinations and treatment were conducted by an international team of dental therapists and dentists registered with the Health Professions Council of Zambia. Each was a member of the oral health collaborative and participated in program design and standardization. Locally trained oral health community volunteers provided oral health education in the local language during the annual outreach programs and periodically throughout the year.

### Sample size

The sample size of 5,791 consisted of all individuals who sought dental care and received a dental screening exam conducted by an oral health collaborative dental provider during the dental outreach programs conducted between 2007 and 2019.

### Data sources and measurement

*Oral Health Data:* The dental screening exam consisted of intraoral and extraoral head and neck evaluations of hard and soft tissues. Oral health indicators for evaluation were selected from the World Health Organization (WHO) [14] and Association of State and Territorial Dental Directors (ASTDD) [15,16] oral health survey resources. All dental screening information was recorded on paper forms utilizing visual examinations by qualified oral health providers using artificial light, disposable dental mirrors, disposable tongue blades, and 2 × 2 gauze. To minimize potential bias in data collection, all dental providers received an educational session detailing screening exams and documentation procedures prior to the start of each annual program. Oral health service activity from 2007 to 2017 and 2018 to 2019 was recorded during oral health screening and treatment sessions. An interruption occurred in program activity from 2015 to 2016 due to funding issues. COVID-19 prevented the reactivation of the program after 2019. Data spanning dental service activity (2007–2014 and 2018–2019) were deidentified and entered in Microsoft Excel. Demographic variables collected included sex and age. Age was distributed into seven categories: 2–5, 6–11, 12–15, 16–19, 20–39, 40–59, and 60+ years. Oral health variables included pain, presence of dental caries, and treatment urgency scores. Oral pain at the time of the screening was self-reported or reported by a parent or guardian. Untreated caries, defined as the presence of at least one visually cavitated lesion, were reported dichotomously as present or absent. Treatment urgency was recorded as a variable with four ordered categories from least to most urgent: “no obvious problem,” “early dental care,” “urgent care, requiring care within 24 hours,” or “in need of immediate referral for life-threatening emergency of dental or other origin.” Due to the presence of only nine subjects in the latter category, the two most urgent categories were combined into a single category, yielding an ordinal variable with three categories for data analysis.

### Statistical analysis

Descriptive statistics (frequencies and percentages) were calculated. Comparisons of year categories were conducted via the chi-square test for demographic variables, pain, and untreated caries and via the Kruskal-Wallis test for treatment urgency. Multivariable models for pain, untreated caries, and treatment urgency were run using binary logistic regression for pain and untreated caries and ordinal logistic regression for treatment urgency. Odds ratios and corresponding 95% confidence intervals were calculated for the multivariable models. The significance level was set at α = 0.05. Data analysis was performed using SPSS 28 (IBM Corp., Armonk, NY, USA).

## Results

### Description of program milestones

During annual visits, Zambian and US oral health teams provided oral health promotion, education, and prevention programs, as well as oral urgent care, including tooth extractions. At the request of the community, the program incorporated the training of ten local community oral health volunteers to provide oral health education, identify community oral health needs, and assist visiting teams. Under the direction of the medical staff at the Rural Health Post, community oral health volunteers received educational assistance and individual mentoring to qualify for admission to the Dental Training School. At the request of the community oral health volunteers, in collaboration with the Dental Training School, a pipeline program was implemented that provided support for qualified community oral health volunteers to attend the Dental Training School for dental therapy, assisting, or technology training. In addition, the collaboration provided the opportunity for students from Tufts University School of Dental Medicine and the Dental Training School to participate in the annual outreach programs.

In response to community requests and in cooperation with the community and the Health District, the visiting oral health teams engaged in advocacy and the identification of funding collaborations for the installation of two boreholes for clean water and the addition of a maternal child/oral health facility at the community Rural Health Post. The Rural Health Post originally consisted of two small buildings with a one-bed delivery area, a pediatric room, two adult medical rooms, three offices, a pharmacy room, and a storage space. It had no running water. The new addition, completed in 2022, has two dental suites, a delivery room with a four-bed capacity, an antenatal ward with an eight-bed capacity, a postpartum ward with a six-bed capacity, a laundry room, a pharmacy, a storage room, a meeting room, and six bathrooms and showers. Reliable, potable running water was installed during the construction of the new facility. Each room has running water. Currently, solar power provides electrification. The Zambian Ministry of Health designated the expanded facility as a Zonal Health Center, provided an ambulance, and significantly increased the staffing for the new facility based on health needs. One midwife, nine nurses, a data technician, and a public health technician were staff additions to the original clinical officer and nurse. The surrounding zonal health posts refer patients, particularly pregnant women in need of obstetric care. This increased the facility’s catchment area from 14,000 to 43,000 people. Dental services are provided at the Zonal Health Center by an oral health outreach team based in the Health District.

### Quantitative analysis

Quantitative oral health data included information from 5791 subjects at the outreach site. Six hundred and twenty-nine subjects participated in the program in 2007–08, 853 in 2009–10, 1,159 in 2011–12, 1,439 in 2013–14, and 1,711 in 2018–19. Table 1 presents cross-tabulations of sex and age category with year category. The percentage of male participants in the program increased from 37.7% in 2007–08 to 58.2% in 2018–19, with this percentage increasing from each year category to the next except from 2009–10 to 2011–12. The difference between year categories was statistically significant (*p* < 0.001). The percentage of participants in the three youngest age categories (2–5, 6–11, and 12–15 years) increased from 5.4% to 8.4%, 16.7% to 28.0%, and 6.7% to 24.7%, respectively, from 2007–08 to 2018–19, while the percentage of participants in the three oldest age categories (20–39, 40–59, and 60+ years) decreased from 40.6% to 18.0%, 21.8% to 10.1%, and 5.3% to 2.4%, respectively, over the same time period. The difference between year categories was statistically significant (*p* < 0.001).

**Table 1:** Cross-tabulation of sex and age categories with year category.*


		2007–08		2009–10		2011–12		2013–14		2018–19		TOTAL n**
				
		*n*	%	*n*	%	*n*	%	*n*	%	*n*	%	

**Sex**	Male	237	37.7	338	39.6	447	38.6	710	49.9	989	58.2	2721

	Female	392	62.3	515	60.4	711	61.4	714	50.1	710	41.8	3042

	*p*	<0.001										

**Age category**	2–5	34	5.4	33	3.9	80	6.9	143	10.0	142	8.4	432

	6–11	105	16.7	153	18.0	220	19.1	400	28.0	476	28.0	1354

	12–15	42	6.7	129	15.2	149	12.9	243	17.0	420	24.7	983

	16–19	22	3.5	59	6.9	75	6.5	86	6.0	144	8.5	386

	20–39	255	40.6	307	36.1	379	32.9	328	23.0	305	18.0	1574

	40–59	137	21.8	130	15.3	196	17.0	168	11.8	171	10.1	802

	60+	33	5.3	40	4.7	53	4.6	60	4.2	40	2.4	226

	*p*	<0.001										


* Data are presented as frequencies and column percentages. ** All 5,791 subjects had valid data for the year category. A total of 5,763 subjects (99.5%) had valid data for sex, while 28 subjects (0.5%) had missing data. A total of 5,757 subjects (99.4%) had valid data for the age category, while 34 subjects (0.6%) had missing data.

Table 2 presents cross-tabulations of pain, untreated caries, and treatment urgency with year category. The percentage of participants with pain decreased from each year category to the next, starting at 60.9% in 2007–08 and decreasing to 22.0% by 2018–19. The percentage of participants with untreated caries also decreased from each year category to the next, starting at 76.5% in 2007–08 and decreasing to 33.9% by 2018–19. The percentage of participants in the treatment urgency category representing the greatest urgency (“urgent care, requires care in 24 hours” or “in need of immediate referral for a life-threatening emergency of dental or other origins”) decreased from each year category to the next, starting at 48.0% in 2007–08 and decreasing to 24.7% in 2018–19. Conversely, the percentage of participants in the category representing the least urgency (“no obvious problem”) increased from each year category to the next, starting at 22.1% in 2007–08 and increasing to 52.4% in 2018–19. The difference between year categories was statistically significant for each of the three outcome variables (pain, untreated caries, and treatment urgency; *p* < 0.001).

**Table 2:** Cross-tabulation of pain, untreated caries, and treatment urgency with year category.*


		2007–08		2009–10		2011–12		2013–14		2018–19		TOTAL n**
				
		*n*	%	*n*	%	*n*	%	*n*	%	*n*	%	

**Pain**	Yes	379	60.9	485	59.1	439	40.1	450	31.4	363	22.0	2116

	No	243	39.1	336	40.9	657	59.9	981	68.6	1285	78.0	3502

	p	<0.001										

**Untreated caries**	Yes	456	76.5	528	63.3	660	58.5	603	42.5	568	33.9	2815

	No	140	23.5	306	36.7	468	41.5	816	57.5	1106	66.1	2836

	*p*	<0.001										

**Treatment urgency**	No obvious problem	132	22.1	294	35.5	514	45.1	734	51.5	890	52.4	2564

	Early dental care	179	29.9	146	17.6	181	15.9	280	19.6	387	22.8	1173

	Urgent care/In need of immediate referral	287	48.0	389	46.9	445	39.0	412	28.9	420	24.7	1953

	*p*	<0.001										


* Data are presented as frequencies and column percentages. ** All 5,791 subjects had valid data for the year category. A total of 5,618 subjects (97.0%) had valid data for pain, while 173 subjects (3.0%) had missing data. In addition, 5,651 subjects (97.6%) had valid data for untreated caries, while 140 subjects (2.4%) had missing data. A total of 5,690 subjects (98.3%) had valid data for treatment urgency, while 101 subjects (1.7%) had missing data.

Table 3 presents the results of the multivariable model with pain as the outcome variable. The association between year category and pain was statistically significant (*p* < 0.001) when adjusting for sex and age. When using the 2007–08 year category as the reference category to which the other year categories were compared, the odds ratio for 2009–10 exceeded 1 (OR = 1.37), and the entire range of its 95% confidence interval was above 1 (95% CI: 1.05–1.79), indicating a statistically significantly greater tendency for pain in 2009–10 compared with 2007–08 when adjusting for sex and age category. However, the odds ratios for 2011–12, 2013–14, and 2018–19 were all less than 1 (OR = 0.41, 0.46, and 0.31, respectively), and the entire ranges of their 95% confidence intervals were below 1 (95% CI: 0.32–0.52, 0.36–0.58, and 0.24–0.39, respectively), indicating significantly less pain in 2011–12, 2013–14, and 2018–19 compared with 2007–08 when adjusting for sex and age category. The comparison of males and females in terms of pain was also significant (*p* = 0.004); when using males as the reference category for sex, the odds ratio and entire range of the 95% confidence interval for females were above 1 (OR = 1.23, 95% CI: 1.07–1.42), indicating significantly greater pain for females when adjusting for year category and age category. Finally, the difference between age categories was significant (*p* < 0.001); when using the 2–5 age category as the reference category, all other categories had odds ratios and 95% confidence intervals entirely above 1, indicating that the latter categories exhibited significantly greater pain than the 2–5 category when adjusting for year category and sex.

**Table 3:** Results of the multivariable model with pain as the outcome variable and year category, sex, and age category as the explanatory variables (*n* = 5562*).


		OR (95% CI)	*p*

**Year category**	2007–08**	1	<0.001

	2009–10	1.37 (1.05–1.79)	

	2011–12	0.41 (0.32–0.52)	

	2013–14	0.46 (0.36–0.58)	

	2018–19	0.31 (0.24–0.39)	

**Sex**	Male**	1	0.004

	Female	1.23 (1.07–1.42)	

**Age category**	2–5**	1	<0.001

	6–11	2.19 (1.33–3.61)	

	12–15	2.65 (1.60–4.41)	

	16–19	6.32 (3.73–10.70)	

	20–39	38.07 (23.60–61.42)	

	40–59	55.95 (34.16–91.65)	

	60+	45.22 (26.07–78.42)	


* For this model, data from 96.0% of subjects were included (i.e., 4.0% of subjects had missing data). ** Reference category.

Table 4 presents the results of the multivariable model with untreated caries as the outcome variable. The association between year category and untreated caries was statistically significant (*p* < 0.001) when adjusting for sex and age. When using the 2007–08 year category as the reference category, the odds ratios and entire ranges of the 95% confidence intervals for all other year categories were less than 1, indicating significantly fewer untreated caries in all other year categories compared with 2007–08 when adjusting for sex and age category. Sex was not a significant explanatory variable in the model (*p* = 0.579), whereas age category was significant (*p* < 0.001). When using the 2–5 age category as the reference category, all other categories had odds ratios and 95% confidence intervals entirely above 1, indicating that these other categories had significantly greater untreated caries than the 2–5 category when adjusting for year category and sex.

**Table 4:** Results of the multivariable model with untreated caries as the outcome variable and year category, sex, and age category as the explanatory variables (*n* = 5597*).


		OR (95% CI)	*p*

**Year category**	2007–08**	1	<0.001

	2009–10	0.63 (0.47–0.83)	

	2011–12	0.50 (0.38–0.65)	

	2013–14	0.32 (0.25–0.42)	

	2018–19	0.25 (0.19–0.32)	

**Sex**	Male**	1	0.579

	Female	1.04 (0.91–1.19)	

**Age category**	2–5**	1	<0.001

	6–11	1.99 (1.47–2.70)	

	12–15	1.70 (1.23–2.34)	

	16–19	2.75 (1.93–3.93)	

	20–39	16.67 (12.31–22.58)	

	40–59	46.93 (32.58–67.60)	

	60+	41.77 (25.23–69.17)	


* For this model, data from 96.6% of subjects were included (i.e., 3.4% of subjects had missing data). ** Reference category.

Table 5 presents the results of the multivariable model with treatment urgency as the outcome variable. The association between year category and treatment urgency was statistically significant (*p* < 0.001) when adjusting for sex and age. When using the 2007–08 year category as the reference category, the 95% confidence intervals of the odds ratios for 2009–10 and 2018–19 contained 1, indicating no significant difference between the latter year categories and the 2007–08 category when adjusting for sex and age category. However, the odds ratios and entire ranges of the 95% confidence intervals for the 2011–12- and 2013–14-year categories were less than 1, indicating significantly lower treatment urgency for the 2011–12 and 2013–14 categories compared with 2007–08. Sex was not a significant explanatory variable in the model (*p* = 0.089), whereas age category was significant (*p* < 0.001). When using the 2–5 age category as the reference category, all other categories had odds ratios and 95% confidence intervals entirely above 1; hence, these other categories had significantly greater treatment urgency than the 2–5 category when adjusting for the year category and sex.

**Table 5:** Results of the multivariable model with treatment urgency as the outcome variable and year category, sex, and age category as the explanatory variables (*n* = 5630*).


		OR (95% CI)	*p*

**Year category**	2007–08**	1	<0.001

	2009–10	1.13 (0.90–1.42)	

	2011–12	0.64 (0.51–0.79)	

	2013–14	0.72 (0.58–0.89)	

	2018–19	0.85 (0.69–1.05)	

**Sex**	Male**	1	0.089

	Female	1.11 (0.99–1.24)	

**Age category**	2–5**	1	<0.001

	6–11	1.71 (1.29–2.27)	

	12–15	2.03 (1.52–2.71)	

	16–19	3.67 (2.65–5.07)	

	20–39	30.16 (22.72–40.03)	

	40–59	72.84 (53.12–99.87)	

	60+	96.55 (62.34–149.54)	


* For this model, data from 97.2% of subjects were included (i.e., 2.8% of subjects had missing data). ** Reference category.

## Discussion

This study exemplified the use of demographic and oral health variables to evaluate the progress of an annual oral health outreach program offering oral health promotion, education, prevention, and dental services in rural Zambia. A single-site analysis revealed increasing participation and changing demographics among the program’s participants over time, suggesting varying demand patterns for oral health services in the community across different years. A key demographic finding was the rise in the number and percentage of participants in the three youngest age categories (2–5, 6–11, and 12–15 years) from 2007–08 to 2018–19. The present data demonstrated a notable rise in male participation in the program, whereas female involvement remained consistent. This suggests that the program successfully expanded its reach to a broader audience as it matured. Significant differences were observed between year categories in terms of pain, untreated caries, and treatment urgency in both bivariate and multivariable analyses. When adjusting for sex and age, pain was significantly lower in the three most recent year categories than in the initial 2007–08 category. Untreated caries were significantly lower in all more recent year categories than the 2007–08 category, and treatment urgency was significantly lower for the 2011–12 and 2013–14 categories compared to the 2007–08 category. These findings align with health awareness and prevention-seeking behavior among the program’s participants. Increased participation in the oral health program and improved oral health measures are also consistent with community-based oral health promotion efforts [17,18] and the motivating factor of receiving toothbrushes and fluoridated toothpaste for at-home care.

The multivariable analysis also allowed for insights into the associations between sex and age category with the outcome variables. When adjusting for the year category and age category, female participants reported significantly more pain than males. Extensive research in other fields has shown similar results and suggested that sex-based differences in pain are multifactorial [19]. Although not well understood, participants in higher age categories exhibited more pain, untreated caries, and treatment urgency when adjusting for year category and sex. These findings support the idea that the oral health outreach program would benefit from a better understanding of the sex-based etiology of oral pain as well as barriers to maintaining oral health among older individuals.

Although we were unable to assess the direct impact of the community oral health volunteer program on program outcome assessments, the oral health volunteer program provided oral health education and supported community utilization of oral health services throughout the entire study period. Prior to the development of the oral health collaborative in 2006, oral health practices in the community were mainly based on traditional cultural beliefs. The curriculum for community oral health volunteers blended traditional cultural oral health information with dental practice recommendations by the oral health collaborative. This was implemented in 2007 and emphasized the relationship between oral health and overall health, effective daily homecare practices, the impact of diet on oral health, the importance of the primary dentition, and the benefit of early oral disease detection for people of all ages. To expand the reach of the volunteers, the collaborative provided bicycles, raincoats, and oral health educational materials for the volunteers to expand oral health educational programs targeting school systems, women’s groups, church organizations, and community functions.

Assessment tools for the evaluation of programs in dynamic populations in under-resourced environments are challenging at best. The results of this study contribute to the limited information available regarding DAO program design and evaluation. While acknowledging the robust nature of the analysis, it is critical to address certain contextual constraints. The range of variables incorporated within the dataset was less extensive than desired and limited to those individuals who self-identified the need for dental care. The dataset did not contain information regarding variables such as education level, income, or dietary habits. This absence, while not diminishing the value of the program assessment data, could introduce nuanced confounding elements into the interpretation.

This multidisciplinary community-engaged dental outreach program resulted in improved trends in oral health and oral health–seeking behavior. Leveraging local, national, and international resources resulted in clean water initiatives and the development of an integrated maternal child and oral health facility. Emphasizing community-identified needs and fostering collaboration with community partners led to sustainable, locally available oral health and health resources delivered by local providers. However, the program design and outcomes are context-specific to the community described in this manuscript, and the valuable insights gained from this long-term collaboration help to create an information repository regarding oral health outreach design. The lessons learned have the potential to inform the development of dental aid outreach efforts that improve oral health outcomes by empowering communities to autonomously manage oral health care for their populations [20].

## Conclusion

Subjects participating in a community-driven oral health education and care program at a site in rural Zambia exhibited an increased interest in asymptotic non-urgent oral health services over time. Participation in the program increased across the year categories. Its demographic patterns changed over time, indicating a trend toward increased demand for oral health services for those not in pain, males, and individuals in younger age categories. Collaboration with community stakeholders led to sustainable efforts for improved clean water sources as well as locally provided maternal, child, and oral health care.
